# Ralph C. Purnell

**Published:** 1887-08

**Authors:** 


RALPH C. PURNELL, M. D., D. D. S., died July 26th, 1887. Dr. Purnell, whose sad death it devolves upon us to record, was a young man of sterling qualities and in the full vigor of health. We can scarcely realize that one, whose face had become so familiar at the University of Maryland during the past three years, has been so suddenly cut off in the prime of life. Dr. Purnell was a young man of fine social qualities and a general favorite with all who knew him, and his sad death will not only be a shock, but a matter of deep regret to his many friends in this city, where he has resided during his studentship at the University of Maryland. He was a graduate of the Dental Department of the University of Maryland of the class of 1886, and in March last, having taken the graded course, received the degree of Doctor of Medicine in the class of 1887 of the University of Maryland School of Medicine. In March last he was appointed Assistant Demonstrator in the Dental Department for the session of 1887-88. The following account of his death appeared in the daily *American*, of this city:

"Dr. Ralph Purnell, of Snow Hill, Md., was drowned yesterday, July 26th. The doctor, in company with his young cousins, Sidney Burroughs and Thomas Collins, were bathing in the surf at Scott's Beach, near here, when Dr. Purnell, who was but a poor swimmer, was carried beyond his depth by the undertow, and becoming fatigued, called loudly for help. In response to his cries, young Collins made an effort to help him, but as soon as Collins reached him the doctor clasped him tightly in his arms. Collins breaking loose, held out one foot and told the doctor to take hold of his foot and he would tow him to shore. This the doctor did, and immediately started to climb upon Collins again. Fortunately for Collins, a huge wave separated them, when Dr. Purnell immediately disappeared, and was seen no more for about an hour, when his lifeless body was found about a quarter of a mile from where he was drowned. Mr. Trump, of Wilmington, Delaware, went in the surf, and finally succeeded in finding the body, which was brought home last night."

---

